# The complete mitochondrial genome of *Nasutitermes tiantongensis* Zhou et Xu, 1993 (Isoptera: Termitidae: Nasutitermitinae)

**DOI:** 10.1080/23802359.2022.2161837

**Published:** 2023-01-15

**Authors:** Kexin Qian, Haihong Chen, Jiaojuan Qian, Qijuan Gu, Dayu Zhang

**Affiliations:** a College of Advanced Agricultural Sciences, Zhejiang A&F University, Hangzhou, Zhejiang Province, China; b Institute of Ningbo Termite Control, Ningbo, Zhejiang Province, China

**Keywords:** *Nasutitermes tiantongensis*, mitochondrial genome, phylogenetic tree

## Abstract

*Nasutitermes tiantongensis* belongs to Nasutitermitinae and its mitochondrial genome was determined in this study. It consisted of 13 PCGs, 22 tRNAs, 2 rRNAs, and an A + T-rich control region, and its length was 15824 bp. The phylogenetic analysis suggested that the genus *Nasutitermes* was not monophyletic, and *N. tiantongensis* formed a sister group with *Bultitermes laticephalus.* The mitochondrial genome of *N. tiantongensis* provides a resource for evolutionary analysis within Nasutitermitinae.

Nasutitermitinae is a large subfamily of Termitidae, and of considerable economic importance (De Faria Santos et al. 2017). *Nasutitermes* belongs to the subfamily Nasutitermitinae, the genus status of many species within this subfamily is uncertain, and the species in the genus *Nasutitermes* is also complex and needed to be reclassification (Inward et al. 2007). Considering the complexity of the subfamily Nasutitermitinae, so we study their mitochondrial genomes of these species in this subfamily. *Nasutitermes tiantongensis* was first mentioned in 1993 (Zhou and Xu 1993). To date, there is no available information for the mitochondrial genome of *N. tiantongensis*. In this study, we firstly reported the complete genome sequence of this species.

Specimens were collected from Ningbo, China (29.86°N, 121.54°E) and deposited at College of Advanced Agricultural Sciences, Zhejiang A&F University (Contact person: Zhang Dayu, and email: zhangdayu@zafu.edu.cn) under the voucher number NH-03. Total genomic DNA was extracted from the heads of 10 worker termites using FastPure Cell/Tissue DNA isolation Mini Kit (Vazyme, Nanjing, China) following the manufacturer’s instructions. Sequencing library was constructed after DNA extraction (Cloudna Technology, Beijing, China), and paired-end reads were sequenced using HiSeq XTen PE 150 of Illumina. The sequences were assembled and annotated using the MitoZ software (Meng et al. 2019). The total mitochondrial genome of *N. tiantongensis* was 15,824 bp in size, including 13 protein-coding genes (PCGs), 22 transfer RNA (tRNAs) genes, 2 ribosomal RNA (rRNAs) genes and a control region located between *rrnS* and *trnI* gene, and its gene arrangement was consistent with other termites (Lee et al. 2017). The minority strand (N-strand) encodes 14 genes (*nad1, nad4, nad4L, nad5, trnQ, trnC, trnY, trnF, trnH, trnP, trnL1, trnV, rrnS* and *rrnL*), and the other 23 genes are located at the majority strand (J-strand).

The total length of 13 PCGs of *N. tiantongensis* mitochondrial genome was 11,139 bp. All the PCGs were initiated with ATN, among which *ATP8, ATP6, ND3* and *ND6* used ATA as start codons, and the remaining used ATG as start codons. Except for *nad1* with *TAG*, *cox2* and *nad5* with incomplete T as stop codon, the other genes were terminated with TAA. The mitochondrial genome of *N. tiantongensis* had 22 tRNAs with the typical clover-leaf secondary structure, aside from *trnS1* lacking the dihydrouridine (DHU), which was common phenomenon observed in metazoan animals (Garey and Wolstenholme 1989). The *rrnS* was located between *trnL* and *trnV*, and *rrnL* was between *trnV* and control region. The control region was 1019 bp in size and had a high A + T content (71.93%).

The phylogenetic tree based on nucleotide sequences of all PCGs from Nasutitermitinae species was constructed to investigate phylogenetic relationship. Maximum likelihood method was used to construct the phylogenetic tree using MEGA X, and the bootstrap value was set as 1000. The results suggested that the genus *Nasutitermes* was not monophyletic, and *N. tiantongensis* formed a sister group with *Bultitermes laticephalus* (Figure 1). Previous phylogenetic analysis inferred from mitochondrial cytochrome oxidase II and 16S rRNA sequences also did not support the monophyly of the genus *Nasutitermes* (Bergamaschi et al. 2007). Phylogenetic relationships based on the analysis of three genes (cytochrome oxidase II, 12S rRNA and 28S rRNA) indicated that *Nasutitermes* was paraphyletic on the estimated cladogram and Nasutitermitinae were monophyletic (Inward et al. 2007). Hence, the genus *Nasutitermes* was paraphyletic. The species in this genus was complex, and worth for further study.

**Figure 1.**
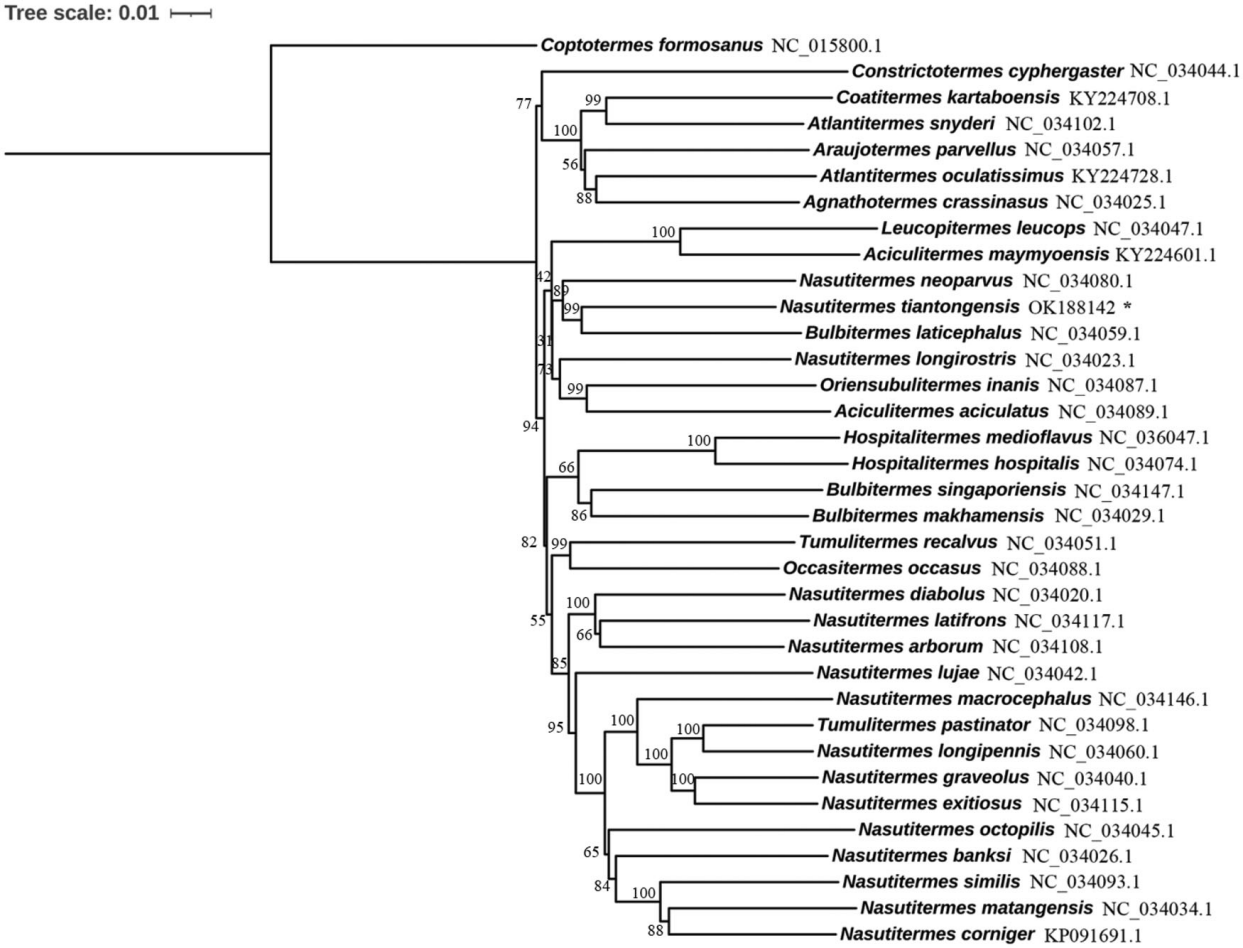
The phylogenetic tree constructed using the nucleotide sequences of 13 PCGs. *Coptotermes formosanus* was set as an outgroup. Leaf names were presented as species names and Genbank accession number. *N. tiantongensis* was labeled with asterisk (*).

## Data Availability

The genome sequence data that support the findings of this study are openly available in Genbank of NCBI at (https://www.ncbi.nlm.nih.gov/) under the accession number OK188142. The associated BioProject, SRA and Bio-Sample number are PRJNA835527, SRR19109539 and SAMN28104798, respectively.
